# Stakeholders’ Interest in and Challenges to Implementing Farm-to-School Programs, Douglas County, Nebraska, 2010–2011

**DOI:** 10.5888/pcd10.130182

**Published:** 2013-12-19

**Authors:** Courtney A. Pinard, Teresa M. Smith, Leah R. Carpenter, Mary Chapman, Mary Balluff, Amy L. Yaroch

**Affiliations:** Author Affiliations: Teresa M. Smith, Amy L. Yaroch, Mary Chapman, Gretchen Swanson Center for Nutrition and University of Nebraska Medical Center, Omaha, Nebraska; Leah R. Carpenter, Gretchen Swanson Center for Nutrition, Omaha, Nebraska; Mary Balluff, Douglas County Health Department, Omaha, Nebraska.

## Abstract

**Introduction:**

Schools are uniquely positioned to influence the dietary habits of children, and farm-to-school programs can increase fruit and vegetable consumption among school-aged children. We assessed the feasibility of, interest in, and barriers to implementing farm-to-school activities in 7 school districts in Douglas County, Nebraska.

**Methods:**

We used a preassessment and postassessment survey to obtain data from 3 stakeholder groups: school food service directors, local food producers, and food distributors. We had a full-time farm-to-school coordinator who was able to engage multiple stakeholders and oversee the development and dissemination of a toolkit. We used descriptive statistics to make comparisons.

**Results:**

Seven food service directors, 5 distributors identified by the food service directors, and 57 local producers (9 completed only the preassessment survey, 16 completed only the postassessment survey, and 32 completed both) completed various components of the assessment. Interest in pursuing farm-to-school activities to incorporate more local foods in the school lunch program increased during the 2-year project; mean interest in purchasing local foods by food service directors for their districts increased from 4.4 to 4.7 (on a scale of 1 to 5).

**Conclusion:**

Implementing farm-to-school programming in Douglas County, Nebraska, is feasible, although food safety and distribution is a main concern among food service directors. Additional research on feasibility, infrastructure, and education is recommended.

## Introduction

Despite recommendations of the 2010 Dietary Guidelines for Americans to consume 5 to 13 servings of fruits and vegetables daily to reduce risk of cardiovascular disease and obesity, many school-aged children in the United States fail to meet recommendations (1,2). Few children (17.6% male, 19.8% female) and adolescents (37.0% male, 28.3% female) report eating the recommended 5 servings of fruits and vegetables daily in a national sample, and in Douglas County, Nebraska, even fewer (<10%) children meet recommendations (3,4). Schools are uniquely positioned to influence the dietary habits of children (5), because they typically provide 2 daily meals (6). Farm-to-school (F2S) programs can increase fruit and vegetable consumption among school-aged children (7,8).

The number of F2S programs is growing across the United States, increasing from 400 participating schools in 2003 (9) to almost 10,000 in 2012 (10). The primary focus of F2S programs is to connect schools with local farms to incorporate healthful foods into school cafeterias, improve student nutrition, provide health and agricultural education, and support local producers (11). Many F2S programs include curricula such as experiential education, taste testing, and farm tours (12). Case studies of school programs suggest that incorporating local foods into school meals increases fruit and vegetable consumption and school meal participation (7,8). In addition, food service directors (FSDs) report that they are motivated to procure from local farmers to support their community (13). However, few community-based evaluations of F2S programs, particularly those that address the logistics and feasibility of implementation and procurement, have been conducted.

We sought to build the capacity for F2S programs in Douglas County schools by developing relationships necessary for procurement of local foods and providing a toolkit aimed at 3 stakeholder groups: FSDs, local food producers, and food distributors. We developed and conducted surveys with these stakeholders to evaluate the process of implementing a new F2S program. 

## Methods

The Douglas County F2S program focused on procurement, encouraging FSDs to purchase foods from local producers. This project was funded as part of the Centers for Disease Control and Prevention Communities Putting Prevention to Work, a 2-year grant awarded to the Douglas County Health Department (14). Preassessment occurred from June through September 2010, and postassessment occurred from September through December 2011. All evaluation procedures were approved by the University of Nebraska Medical Center Institutional Review Board.

All FSDs from the Douglas County school districts (n = 7), distributors identified by FSDs (n = 5), and a convenience sample of local producers (41 preassessment, 48 postassessment) were recruited to complete preassessment and postassessment surveys tailored for each group. Douglas County is located in eastern Nebraska, includes a mix of urban and suburban communities (population = 524,861), and is the most racially/ethnically diverse county in Nebraska (Table 1) (15).

**Table 1:** Characteristics of 7 School Districts in Douglas County, Nebraska

Characteristic	OPS	Millard	Westside	Elkhorn	Ralston	Bennington	DC-West
**No. of schools**	108	35	12	12	8	4	3
**No. of students**	50,461	22,417	5,986	5,896	3,100	1,576	631
**No. of lunches served daily**	34,588	14,610	3,736	3,546	2,014	775	385
**Free and reduced-price lunch,^a^ %**	73.1	16.1	30.8	7.0	46.5	10.2	29.0
**Student race/ethnicity, %**							
White	32.3	83.3	77.6	92.2	65.0	89.0	89.0
Black	26.0	2.9	7.8	3.2	7.0	2.9	7.0
Hispanic	31.4	6.1	6.1	1.5	25.0	3.7	0.5
Other	10.3	7.7	8.5	3.2	3.0	4.5	3.5
**Cost per lunch, $**	1.65	2.45	2.05	2.50	2.20	—	2.32
**Food service staff working status**							
No. who work full time	211	170	50	4	10	6	1
No. who work part time	195	15	17	70	30	7	9

Abbreviation: OPS, Omaha Public Schools; DC, Douglas County; —, not calculated.

a Free and reduced-price lunch refers to the proportion of students who receive lunch at a reduced price or for free on the basis of low-income eligibility criteria.

Engaging community members and stakeholders is a critical step to promote F2S, which was a new idea for this community and required capacity building. In early 2010, we identified FSDs and conducted in-person meetings with them to gain a better understanding of the operations of each district. We also met with local producers to gauge interest in and capacity for selling to schools. We worked directly with the stakeholders to minimize perceived barriers and provide opportunities for developing necessary relationships and developed an online toolkit (toolkit.centerfornutrition.org) for use by the 3 stakeholder groups. This toolkit is comprehensive, discusses general F2S concepts, and provides information and resources for the stakeholders. Throughout the project, we conducted activities and met several milestones (eg, creating procurement policy, holding stakeholder meetings, and developing and launching a toolkit) to increase procurement of local foods in the 7 school districts.

We administered preassessment and slightly modified postassessment surveys (ie, some items anticipated to remain static were not readministered) to each stakeholder group. Questions were derived from the literature (7) and supplemented as needed to assess key factors related to local food procurement. The postassessment survey determined changes in practices and attitudes that may have occurred among the stakeholders since the initial preassessment survey. Surveys used a 5-point Likert scale (1= strongly disagree to 5 = strongly agree). The FSD survey (49 items) assessed basic information on meal programs, facility capacity, food purchasing, local food practices, attitudes toward local food, perceived barriers, and interest in participating in F2S programming. The producer survey (35 items) assessed the type of production, selling practices, willingness to participate in F2S, and insurance and safety practices. The distributor survey (20 items) assessed service area and distribution practices, sales to schools, local food practices, and willingness to participate in F2S programs.

Surveys were distributed by multiple methods (ie, conducted online via a survey collection program, mailed, hand delivered, and conducted over the telephone) to reach all stakeholders and took approximately 30 to 45 minutes to complete. For both the FSDs and the distributors, survey responses were matched between preassessment and postassessment. For the producers, responses were matched (with a reduced sample, n = 32); otherwise, preassessment data are indicated.

Data were analyzed using SPSS version 19.0 (SPSS, Chicago, Illinois). Descriptive statistics were obtained to examine the distribution of the responses and to calculate frequencies, means, and standard deviations (SDs).

## Results

### Food service directors

All FSDs completed the surveys. School districts varied in size, ranging from 108 schools (including elementary, middle, and high schools) and 50,461 students to 3 schools and 631 students (Table 1). The average cost for lunch was $2.20. Some districts had many salad bars (n = 35), and others had only a few. Kitchen environments of districts varied; FSDs indicated that most kitchens operated on “semi-prepared” ability, meaning that they had satellite kitchen systems in which meals are shipped within districts daily.

At preassessment, FSDs generally agreed that their facilities had capacity to handle serving local foods in schools and had flexibility in producing delivery schedules (mean score, 3.9; SD = 1.0) (Table 2). At postassessment, they indicated lesser agreement on capacities (mean score, 3.4; SD = 1.1), although they maintained agreement in facilities’ capacity. FSDs reported greater agreement that they had adequate dry storage at postassessment than they did at preassessment.

**Table 2:** Interest in and Barriers to Farm-to-School Programming by School Food Service Directors (n = 7) in Douglas County, Nebraska, 2010–2012

Item	Preassessment Value^a^ (June–September 2010)	Postassessment Value^a^ (September–December 2011)	Difference^a^^,^^b^
**Purchasing intentions and reporting, n (%)**			
Did you purchase any locally grown food directly from a grower during this school year?	0	2 (28.6)	2
Do you plan to purchase any locally grown products during next school year?	1 (14.3)	6 (85.7)	5
**Facility capacity, mean score (SD)**			
My facilities have adequate cold storage space to accommodate an increased use of fresh fruits and vegetables.	3.9 (1.4)	3.6 (1.1)	−0.3
My facilities have adequate dry storage space to accommodate an increased use of fresh stored fruits and vegetables.	3.7 (1.2)	4.0 (1.0)	0.3
My facilities have adequate preparation space for fresh fruits and vegetables.	3.7 (1.2)	3.0 (1.3)	−0.7
My facilities are equipped to prepare whole fruits and vegetables (eg, supply of knives, food processors, wedgers, peelers, slicers).	4.4 (0.5)	3.7 (0.8)	−0.7
The food service staff at my facilities are well trained to prepare fresh fruits and vegetables.	4.6 (0.5)	3.4 (0.8)	−1.2
Time limits my facilities’ abilities to use more whole fruits and vegetables in lunches.	3.0 (1.4)	2.9 (1.4)	−0.1
**Attitudes toward local foods, mean score (SD)**			
Supports the local economy.	—	4.3 (0.5)	—
Supports Nebraska farms/businesses.	4.6 (0.5)	4.1 (0.4)	−0.5
Is a good public relations strategy.	4.6 (0.5)	4.3 (0.5)	−0.3
Increases student consumption/awareness of fresh fruits and vegetables.	4.0 (1.2)	4.0 (0.6)	0
Provides higher-quality/fresher products.	3.6 (1.1)	3.3 (1.0)	−0.3
Responds to public demand.	4.3 (0.8)	3.7 (0.5)	−0.6
Supports less use of pesticides.	3.1 (0.9)	2.7 (0.5)	−0.4
**Barriers to purchasing locally, mean score (SD)**			
Finding farmers to purchase from directly.	3.7 (1.8)	3.5 (1.2)	−0.2
Liability/farmer compliance with food safety and food handling standards.	4.0 (1.8)	3.7 (1.1)	−0.3
The timing and frequency of backdoor deliveries.	2.6 (1.9)	3.0 (1.3)	0.4
The added time needed to prepare and handle fresh produce.	2.1 (2.0)	2.7 (1.4)	0.6
Distributor does not offer local options.	2.7 (2.1)	3.1 (1.2)	0.4
Product quality concerns.	1.3 (1.6)	2.5 (0.8)	1.2
Too many other initiatives to juggle.	1.3 (1.7)	2.7 (1.4)	1.4
Fitting local food into budgets.	2.0 (1.2)	2.9 (1.1)	0.9
The need for multiple orders and invoices.	1.6 (1.4)	2.0 (0)	0.4
Difficulty working seasonal produce into menus.	1.7 (1.1)	2.7 (1.4)	1.0
**Interest in conducting farm to school activities, mean score (SD)**			
Asking current vendor(s) to sell local farm products.	3.6 (1.3)	3.9 (1.1)	0.3
Highlighting locally grown foods on printed/online menus.	4.4 (0.8)	4.6 (0.5)	0.2
Planning menus around seasonal availability of local products.	4.6 (0.5)	4.4 (0.5)	−0.2
Buying and highlighting local products each month	4.6 (0.5)	4.4 (0.8)	−0.2
Hosting local food meals or events each school year (eg, Fall Harvest Festival).	3.7 (1.0)	3.9 (1.2)	0.2
Serving local foods on a limited or pilot basis, such as at only 1 school.	3.7 (1.0)	3.7 (1.5)	0
Educating students about local food with educational materials and events (eg, taste testings, farmer visits)	4.0 (0.8)	4.3 (1.0)	0.3

a All values are reported as Likert scores (1 = strongly disagree to 5 = strongly agree) unless otherwise indicated.

b Some differences calculate incorrectly due to rounding.

**Figure 1:**
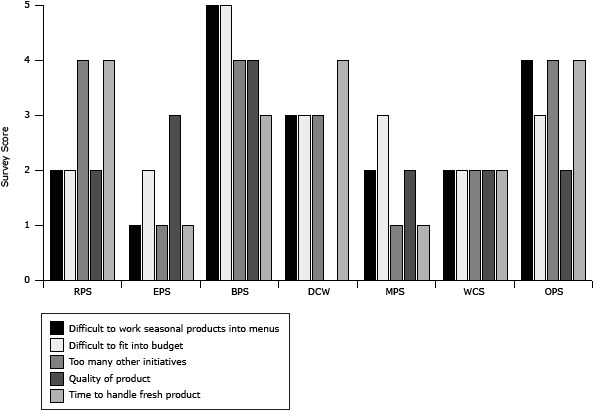
Food service directors’ barriers to procuring local food, responses to preassessment survey, Douglas County, Nebraska, July through September 2010. Surveys used a 5-point Likert scale (1 = strongly disagree to 5 = strongly agree). Dashes indicate missing data. Abbreviations: RPS, Ralston Public Schools; EPS, Elkhorn Public Schools; BPS, Bennington Public School; DCW, Douglas County West; MPS, Millard Public Schools; WCS, Westside Community Schools; OPS, Omaha Public Schools. **Table:** 

**Figure 2:**
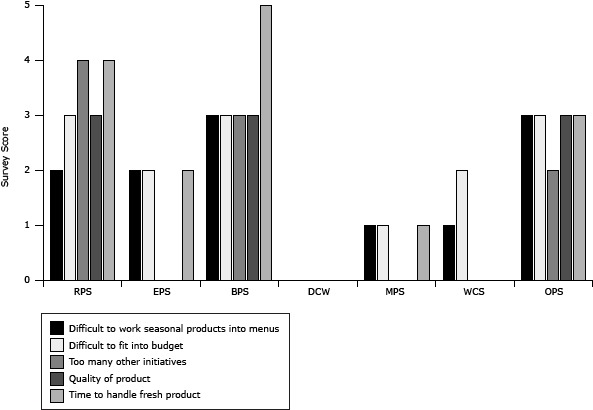
Food service directors’ barriers to procuring local food, responses to postassessment survey, Douglas County, Nebraska, September through December 2011. Surveys used a 5-point Likert scale (1 = strongly disagree to 5 = strongly agree). Dashes indicate missing data. Abbreviations: RPS, Ralston Public Schools; EPS, Elkhorn Public Schools; BPS, Bennington Public School; DCW, Douglas County West; MPS, Millard Public Schools; WCS, Westside Community Schools; OPS, Omaha Public Schools. **Table:** 

In general, FSDs’ willingness to participate in F2S improved from preassessment to postassessment; small increases were seen in interest in purchasing local foods for their district (from 4.1 [SD = 0.8] to 4.2 [SD = 0.9]) and willingness to pay higher prices for locally produced foods (from 3.4 [SD = 0.5] to 3.5 [SD = 0.8]). All FSDs added a signed memorandum, stating a preference for local whenever possible, to their operating procedures. In terms of attitudes toward the benefits of local foods, though agreement decreased slightly at postassessment, respondents generally indicated agreement that local foods supported the economy, increased students’ fruit and vegetable consumption, responded to public demand, and provided higher-quality products. FSDs indicated that overall barriers to purchasing local foods increased slightly, although the ability to find farmers to purchase from and liability and farmer compliance with food safety and handling standards decreased. Across school districts, the response to barriers varied; smaller school districts reported more increases in barriers than did larger school districts (Figure 1, Figure 2). FSDs reported an overall increase in interest in conducting some F2S activities, including asking current vendors to sell local products, highlighting local foods on menus, and hosting local food events (eg, fall harvest festivals). Attitudes toward F2S activities decreased slightly for the following: planning menus around seasonal products each month and buying and highlighting local products each month.

Barrier	RPS	EPS	BPS	DCW	MPS	WCS	OPS

	Survey Score						
Difficult to work seasonal products into menus	2	1	5	3	2	2	4
Difficult to fit into budget	2	2	5	3	3	2	3
Too many other initiatives	4	1	4	3	1	2	4
Quality of product	2	3	4	—	2	2	2
Time to handle fresh product	4	1	3	4	1	2	4

Barrier	RPS	EPS	BPS	DCW	MPS	WCS	OPS

	Survey Score						
Difficult to work seasonal products into menus	2	2	3	—	1	1	3
Difficult to fit into budget	3	2	3	—	1	2	3
Too many other initiatives	4	—	3	—	—	—	2
Quality of product	3	—	3	—	—	—	3
Time to handle fresh product	4	2	5	—	1	—	3

### Local producers

Forty-one local producers responded to the survey in 2010 and 48 in 2011. At preassessment, producers reported producing a range of foods, including vegetables (53%), meat (33%), and dairy (27%). Respondents were from 34 cities in 22 counties in Nebraska (86% in 2010; 89% in 2011), Iowa (12% in 2010; 11% in 2011), and South Dakota (2% in 2010). Of the 57 producers who responded to the survey, 15.8% (n = 9) completed only the preassessment survey, 28.1% (n = 16) completed only the postassessment, and 56.1% (n = 32) completed both and were included in the main pre–post analysis. At preassessment, 24% of producers reported having hoop houses (ie, greenhouse alternatives made of a plastic roof and flexible piping that provide season extension to farmers in colder climates), and at postassessment 55% of producers reported having hoop houses.

Some assessment was completed by the 32 producers because they completed both the preassessment and the postassessment. Among the 32 producers who completed both the preassessment and postassessment, most reported willingness at both preassessment (mean score = 4.7; SD, 0.7) and postassessment (mean score = 4.2; SD, 1.1) to sell to local schools. At postassessment, the number of producers who reported selling to other institutions (eg, hospitals) increased (from 77% to 79%). Most producers reported having a vehicle available for delivery (96% in 2010, 84% in 2011). However, fewer had cold storage (79% in 2010, 58% in 2011) or a staff driver (39% in 2010, 32% in 2011). High levels of willingness to participate in F2S activities were reported at both preassessment and postassessment (mean score, range = 3.7–4.8), with slight decreases overall. Producers expressed willingness to offer taste testing to staff and children, lead guided tours of their farm for staff and children, visit schools or classrooms and speak about their farm products and how they are grown, and join a consortium of producers (Table 3). At postassessment, 78% (n = 32) had liability insurance, averaging $1,432,143 (range = $200,000–$5,000,000).

**Table 3:** Views of Producers and Distributors About Farm-to-School Programming, Douglas County, Nebraska, 2010–2012

Item	Preassessment Score, **a** Mean (SD) (June–September 2010)	Postassessment Score, **a** Mean (SD) (September–December 2011)	Difference^a^^,^ b
**Willingness of producers (n = 32) to participate in farm-to-school activities**			
Taste testing to food service staff.	4.2 (1.1)	3.8 (1.1)	−0.4
Taste testing to children.	4.1 (1.2)	3.7 (1.1)	−0.4
Guided tours on their farm for the staff.	4.8 (0.6)	4.0 (1.2)	−0.8
Guided tours on their farm for the students.	4.2 (1.5)	3.9 (1.2)	−0.3
Visit schools or classrooms.	4.5 (1.2)	4.0 (1.0)	−0.5
Join a consortium of producers.	4.2 (1.2)	3.7 (1.2)	−0.5
**Willingness of distributors (n = 5) to participate in farm-to-school activities**			
How willing would you be to change some of your practices to focus more on local foods?	4.4 (1.1)	3.8 (0.8)	−0.6
**My company would buy more local foods if . . .**			
There were greater availability of products throughout the whole year.	3.2 (1.7)	4.8 (0.4)	1.6
Producers had enough quantity products.	3.2 (1.7)	4.8 (0.4)	1.6
All producers met minimum food safety standards.	3.2 (1.7)	4.2 (1.3)	1.0
Producers used a central packing house or food hub for product pick-ups.	3.2 (1.7)	3.4 (1.3)	0.2
Local producer pricing was competitive.	3.2 (1.7)	4.8 (0.4)	1.6
Processed foods met customer specifications (eg, sizing, packaging).	3.7 (0.6)	4.6 (0.6)	0.9
There was greater demand for local products.	3.7 (1.5)	4.4 (0.6)	0.7

a Calculated from responses to a 5-point Likert scale (1 = strongly disagree to 5 = strongly agree).

b Some differences calculate incorrectly due to rounding.

Some assessment was completed among 41 producers who reported at preassessment (32 of these also completed the postassessment). At preassessment (n = 41), only 7% of the producers responded affirmatively to having Hazard Analysis and Critical Control Points and only 7% reported being certified for Good Agricultural Practices — both of which are food safety components that some schools require. In addition, 78% of the respondents reported having insurance for their general operations and insurance that would cover children and staff on field trips; however, 15% of all surveyed responded that they would not be willing to host field trips.

Several barriers to selling to schools were identified. At postassessment, the barriers with the highest agreement across all producers included inability to provide product during the entire school year, inability to produce sufficient volume of produce, and inability to provide the most competitive price.

### Distributors

All 5 of the distribution company representatives identified by the FSDs completed the preassessment and postassessment surveys. At preassessment, distributors reported that, on average, 13% of their business went to schools; at postassessment, this was reduced to 10%. At preassessment and postassessment, all 5 distributors reported some local purchasing within the past year from a variety of local producers. At preassessment, 4 of the 5 distributors required producers to have a certain level of liability insurance and food safety standards and meet packing specifications.

At preassessment, distributors reported overall willingness to change some of their practices to focus more on local foods (mean score = 4.4; SD, 1.1), which decreased at postassessment (mean score = 3.8; SD, 0.8; Table 3). Increases occurred from preassessment to postassessment, indicating distributors would buy more local food if it were available year-round, producers had sufficient quantity of products, producers met food safety standards, a local food hub or packing house were available for central pick-ups, local pricing were competitive, local producers met customer specifications, and there were greater demand for local products.

## Discussion

This study aimed to determine the feasibility of F2S in schools in Douglas County, Nebraska, by assessing the needs of, attitudes of, barriers to, and capacity among school FSDs, producers, and distributors regarding procurement of local foods before and after the implementation of a F2S program. Overall, the piloted F2S program demonstrated success and sustainability across all districts. Before the program, no districts were purchasing locally produced food. At postassessment, most districts responded that they planned on purchasing local food during the next school year. Despite the evaluation indicating that small increases in many of the perceived barriers, small decreases in perceptions of facility capacities, and some shifts in attitudes away from willingness to participate in a F2S program among key stakeholders, constructs assessed remained favorable toward F2S activities.

Overall, FSDs’ perceptions of their facilities’ capacities were positive, even with slight reported decreases. They reported having sufficient cold storage, dry storage, preparation space, and equipment for handling whole foods, training for staff to prepare whole fruits and vegetables, and time necessary to use whole fruits and vegetables in lunches. These findings are important, because most districts operate using a satellite kitchen system. After gaining experience with this F2S program, FSDs agreed that they could implement the necessary preparation with current resources. Perceptions of barriers remained low at preassessment and at postassessment. Larger school districts reported more reduction in perceived barriers, whereas smaller school districts reported more increases in perceived barriers. FSDs reported willingness to pay higher prices for local foods, which suggests that cost may not be a major barrier (16). Similarly, a study of FSDs from 7 F2S programs found that FSDs reported that prices associated with local food procurement were acceptable and competitive (16). Findings from another study indicated that top barriers to procuring local foods in schools included seasonal availability and lack of partially processed product (17). FSDs’ willingness to participate in F2S and recognition of the benefits of purchasing local foods increased during the project. Interestingly, the larger school districts were more engaged than the smaller school districts throughout the project with more reported activities such as salad bars, labeling local foods on school menus, and farmers markets at school. The FSDs signed a memorandum that stated a preference for local products whenever possible, establishing an informal policy that was easier to accomplish than establishing larger formal policies.

Producer’s levels of willingness to offer F2S education remained high from preassessment through postassessment. Many producers reported an inability to sell produce to schools year-round as a major barrier. However, incorporating dairy and meat may support year-round participation, as other F2S programs located in cold-weather climates made this a practice (www.farmtoschool.org). In addition, the use of seasonal extension methods (eg, hoop houses are viable options for producers to extend the growing season [18]) increased during the project, further supporting year-round production feasibility. Season extension has been noted as a strategy for producers, given increased demand for local production (19). Producers identified other issues such as liability insurance, which would be important for students engaging in educational activities at local farms. It became apparent throughout this project that there was a lack of infrastructure to support sourcing from local producers. Producer willingness to join a consortium, such as one to sell produce to schools or a food hub, could be a viable way of providing local food to larger institutions, such as schools (20). To expand F2S programs and solidify the links between producers and school programs, it is important to consider the needs and motivations of the producers (21). One community has found that both growers and buyers were supportive of the development of a food hub as a way to increase availability of local foods (18).

Distributors are the link between producers and schools, and few previous F2S studies assessed how this key stakeholder can influence success in local food procurement. Distributors were the most difficult stakeholder to engage throughout this project, which could be explained by findings of a similar study that found that distributors considered budget constraints and the need for large orders to cover overhead costs major barriers to participating in F2S (22). This difficulty in engaging distributers may also be related to an increase in perceived barriers. Specifically, the barriers that increased the most were availability of products year-round and ability to provide competitive pricing, which may be related to the fact that key stakeholders were not previously exposed to F2S. To overcome these barriers, we provided education on season extension, made products available year-round, and specified local products in the bidding procedure. Other communities have shown that engaging distributors to track and promote local purchases is a successful strategy (23). The negative shift in distributor attitudes and perceptions could also be due to first-time exposure to F2S.

Our study has limitations, most of which are related to the self-reported nature of the data collected from stakeholders, which could potentially introduce social desirability and recall bias. Because studying F2S is new, we used surveys that were modified versions of existing surveys, which have not undergone validity and reliability testing. Additionally, convenience sampling used for producers at both preassessment and postassessment resulted in only one-third completing both surveys, potentially introducing bias. 

Despite these limitations, this study adds to the literature, as few F2S programs have evaluated the feasibility of procurement. Additionally, it helped identify barriers and sustainable solutions in a geographic area where F2S has not previously been evaluated. The findings of this study augment evidence of programs designed to increase procurement of healthful foods in school settings through partnership with local stakeholders. The data were collected from a diverse sample of stakeholders from the community, representing all aspects of the school food system continuum. 

These findings and the online toolkit can inform future interventions for communities interested in implementing procurement-based F2S programs. Future studies should explore how enhanced infrastructure, like food hubs, can help FSDs procure more local foods. Enhancing the link between production, distribution, and procurement can strengthen the local food system and ultimately increase consumption of local foods.
